# Skeletal muscle munc18c and syntaxin 4 in human obesity

**DOI:** 10.1186/1743-7075-5-21

**Published:** 2008-07-24

**Authors:** Bryan C Bergman, Marc-Andre Cornier, Tracy J Horton, Daniel H Bessesen, Robert H Eckel

**Affiliations:** 1 Division of Endocrinology, Diabetes, and Metabolism, University of Colorado Denver, Aurora, CO, USA; 2 Section of Nutrition, Department of Pediatrics, University of Colorado Denver, Aurora, CO, USA

## Abstract

**Background:**

Animal and cell culture data suggest a critical role for Munc18c and Syntaxin 4 proteins in insulin mediated glucose transport in skeletal muscle, but no studies have been published in humans.

**Methods:**

We investigated the effect of a 12 vs. 48 hr fast on insulin action and skeletal muscle Munc18c and Syntaxin 4 protein in lean and obese subjects. Healthy lean (n = 14; age = 28.0 +/- 1.4 yr; BMI = 22.8 +/- 0.42 kg/m^2^) and obese subjects (n = 11; age = 34.6 +/- 2.3 yr; BMI = 36.1 +/- 1.5 kg/m^2^) were studied twice following a 12 and 48 hr fast. Skeletal muscle biopsies were obtained before a 3 hr 40 mU/m^2^/min hyperinsulinemic-euglycemic clamp with [6,6-^2^H_2_]glucose infusion.

**Results:**

Glucose rate of disappearance (Rd) during the clamp was lower in obese vs. lean subjects after the 12 hr fast (obese: 6.25 +/- 0.67 vs. lean: 9.42 +/- 1.1 mg/kgFFM/min, p = 0.007), and decreased significantly in both groups after the 48 hr fast (obese 3.49 +/- 0.31 vs. lean: 3.91 +/- 0.42 mg/kgFFM/min, p = 0.002). Munc18c content was not significantly different between lean and obese subjects after the 12 hour fast, and decreased after the 48 hr fast in both groups (p = 0.013). Syntaxin 4 content was not altered by obesity or fasting duration. There was a strong positive relationship between plasma glucose concentration and Munc18c content in lean and obese subjects during both 12 and 48 hr fasts (R^2 ^= 0.447, p = 0.0015). Significant negative relationships were also found between Munc18c and FFA (p = 0.041), beta-hydroxybutyrate (p = 0.039), and skeletal muscle AKT content (p = 0.035) in lean and obese subjects.

**Conclusion:**

These data indicate Munc18c and Syntaxin 4 are present in human skeletal muscle. Munc18c content was not significantly different between lean and obese subjects, and is therefore unlikely to explain obesity-induced insulin resistance. Munc18c content decreased after prolonged fasting in lean and obese subjects concurrently with reduced insulin action. These data suggest changes in Munc18c content in skeletal muscle are associated with short-term changes in insulin action in humans.

## Introduction

Insulin resistance is commonly observed in obese humans. Defects in skeletal muscle insulin signaling have been extensively studied, and much is now known about obesity and Type 2 diabetes which can help explain insulin resistance in these populations. However, the role of alterations in GLUT4 vesicle tethering, docking, and fusion to the plasma membrane in causing insulin resistance is not clear.

Increasing evidence suggests GLUT4 vesicle fusion to the sarcolemma is under tight regulation by the coordinated action of soluble N-ethylmaleimide-sensitive factor attachment protein receptors (SNARE's) on both the GLUT4 vesicle (v-SNARE's) and the inner surface of the target plasma membrane (t-SNARE's) [1]. Vesicle associated membrane protein 2 (VAMP2) is a v-SNARE interacting with Syntaxin 4 in the docking step of GLUT4 translocation. Munc18c modulates interactions required for vesicle fusion [1] and is thought to bind to Syntaxin 4 which prevents interaction with VAMP2 [2], although other models have been proposed [3,4]. Insulin binding to the sarcolemma may remove inhibition of Munc18c either through dissociation or repositioning [5], promoting Syntaxin 4 and VAMP2 interaction, allowing GLUT4 vesicle fusion to the plasma membrane [6].

Animal data suggest a critical role played by the relative proportion of Munc18c and Syntaxin 4 proteins in skeletal muscle. Over expression of Munc18c relative to Syntaxin 4 in skeletal muscle leads to insulin resistance [7,8]. Heterozygous knockout of Syntaxin 4 in mice also results in insulin resistance [9]. Importantly, Spurlin et al. found that the relative abundance of Munc18c/Syntaxin 4 was more influential than the absolute protein content on whole body glucose disposal in transgenic mice [7]. These results have been corroborated in 3T3L1 adipocyte models where increased expression of Munc18c decreased insulin action [6,10-12], or microinjection of Munc18c antibodies to decrease cellular content increased insulin action [11]. No published investigations have examined the potential importance of Munc18c and Syntaxin 4 in human muscle. This study was designed to determine if Munc18c and Syntaxin 4 are present in human skeletal muscle. We tested if the amount of Munc18c and Syntaxin 4 in skeletal muscle differ in obese and lean humans, and if the relative expression changes in response to the acute induction of insulin resistance via fasting.

## Methods

### Subjects

Healthy, sedentary, lean men and women and non-diabetic obese men and women with normal glucose tolerance who were weight stable for at least 6 months were recruited for this study (Table 1). Women were taking oral contraceptives and were studied in the week they were not taking the active pill. This study was approved by the Colorado Multiple Institution Review Board at the University of Colorado Denver and Health Sciences Center.

**Table 1:** Subject Characteristics

	Lean	Obese
N (M/W)	(7/7)	(5/6)
Age (yrs)	28 ± 1.4	34.6 ± 2.3§
Weight (kg)	68.2 ± 3.0	102.7 ± 3.5§
Height (in)	69 ± 1.3	68.3 ± 1.4
BMI (kg/m^2^)	22.8 ± 0.4	35.4 ± 1.3§
% Body Fat	24.5 ± 2.3	42.3 ± 2.5§
Waist Circumference (in)	33.2 ± 1.1	44.1 ± 1.4§
Fasting Glucose (mg/dl)	84.2 ± 1.5	90.1 ± 2.2§
Fasting Insulin (uU/ml)	5.5 ± 0.6	8.3 ± 1.4
2 hour OGTT glucose (mg/dl)	95.6 ± 9.1	120.5 ± 8.1§
12 hr clamp glucose Rd (mg/kg FFM/min)	9.42 ± 1.1	6.25 ± 0.67§
48 hr clamp glucose Rd (mg/kg FFM/min)	3.91 ± 0.42#	3.49 ± 0.31#

Values are means ± SEM. § = significantly different than lean (p < 0.05), # = significantly different than 12 hr (p < 0.05).

### Diet and Fasting

A standardized diet containing 30% fat, 15% protein, and 55% carbohydrate was provided to each subject for 3 days prior to both metabolic studies which were separated by 1–2 months. Subjects remained resident on the General Clinical Research Center (GCRC) during the 12 and 48 hour fasts which were completed in random order. A strict fast was imposed where subjects were only allowed to consume water, ice, and flavored non-caloric carbonated water.

### Metabolic Studies

In the morning after completion of the 12 or 48 hr fast, a baseline blood sample was drawn followed by a primed (4.5 mg/kg) constant (0.03 mg/kg/min) infusion of [6,6-^2^H_2_]glucose. Following two hours of rest and blood sampling, a vastus lateralis muscle biopsy was performed using the Bergstrom needle technique [13]. The muscle biopsies were taken from midway between the greater trochanter of the femur and the patella. The anatomic location and depth of the biopsy was as similar as possible between subjects to minimize variation in muscle fiber composition which varies along the depth and length of the vastus lateralis [14]. During the repeat study, the contralateral leg was used for the muscle biopsy. A hyperinsulinemic-euglycemic clamp was initiated using a descending prime of insulin with a continuous infusion at 40 mU/m^2^/min and was continued for the next 3 hours using the method of DeFronzo et al. [15].

### Tissue Processing

Frozen skeletal muscle samples were weighed, and homogenized on ice using a Kontes glass homogenizer (Kimble/Kontes, Vineland, NJ) in buffer containing 20 mM Tris-HCl, pH 7.4, 150 mM NaCl, 1% IPEGAL, 20 mM NaF,2 mM EDTA, pH 8.0, 2.5 mM NaPP, 20 mM β-Glycero-PO_4_, 10% glycerol, as well as protease (Roche Applied Science, Indianapolis, IN) and phosphatase inhibitors (Sigma, Saint Louis, MO). Samples were agitated at 4°C for 1 hour, then spun at 16,000 g for 15' to pellet insoluble protein. Supernatant was saved, and used to determine protein concentration (Pierce, Rockford, IL).

### Western Blotting

Fifty μg of standard, sample protein, and internal standard were run on an SDS-PAGE 4–15% Tris-HCl gel (BioRad, Hercules, CA), transferred to a PVDF membrane, and blocked with 5% nonfat milk or BSA for 1 hour at room temperature. Primary antibody incubations were performed in 5% nonfat milk or BSA overnight at 4°C, and an HRP conjugated secondary antibody was incubated for 1 hour at room temperature. Enhanced chemiluminescence was used to visualize protein bands of interest. Intensity of protein bands was captured using an AlphaImager 3300 (Alpha Innotech Corp, San Leandro, CA) and quantified using FluorChem software (Alpha Innotech Corp, San Leandro, CA). Protein bands were normalized to GAPDH as a loading control, and then normalized to an internal standard which was run on every gel. Rabbit anti-Munc18c antibodies were a generous gift from Dr. David James, mouse anti-Syntaxin 4 antibodies were purchased from BD Transduction Laboratories (BD Biosciences, Mississauga, ON Canada), GAPDH antibodies were purchased from Santa Cruz Biotechnology (Santa Cruz Biotechnology, Santa Cruz, CA) and secondary antibodies from Chemicon (Chemicon International Inc, Temecula, CA).

### Gas chromatography/mass spectroscopy methods

Glucose isotopic enrichment was measured with the penta-acetate derivative of glucose using standard GC/MS methodology (5890 GC, 5989 MS, Hewlett-Packard) [16,17]. Rates of glucose disappearance (Rd) during the clamp were calculated using a modified Steele equation described by Finegood, et al [18].

### Statistics

Data are presented as mean ± SEM. After the 12 hour fast, muscle biopsies from 9 lean subjects and 6 obese subjects were analyzed, while after the 48 hour fast muscle biopsies from 7 lean subjects and 6 obese subjects were analyzed. Differences between fasting duration and lean and obese groups were analyzed using repeated measures ANOVA with one 2-level within subject factor (12 vs. 48 hr fast), and one 2-level between subjects factor (lean vs. obese) (JMP statistical software, Cary, NC). An alpha level of 0.05 was used throughout. Since this is the first report of Munc18c and Syntaxin 4 in skeletal muscle, we are reporting all correlations significant at the alpha 0.05 level without correcting for multiple comparisons. Correlations were performed to all the metabolic and insulin signaling variables measured in the parent study [16], and the significant relationships reported here. We hope this promotes hypothesis formation, furthering research in this area.

## Results

### Subject Characteristics

Anthropometric data for subjects are reported in Table 1. Outcome variables were similar between genders, so men and women were combined and data are reported as lean and obese only. Per design, lean subjects weighed significantly less (p < 0.05), and had a lower BMI, % body fat, and waist circumference than obese (p < 0.05).

### Insulin Clamp

Data on insulin sensitivity in these subjects has previously been published [16]. Insulin stimulated glucose Rd was significantly greater in lean compared to obese subjects after the 12 hour fast (Table 1). After the 48 hour fast insulin stimulated glucose Rd was significantly lower than the 12 hour fast in lean and obese subjects (p < 0.001). The decrease in glucose Rd from the 12 to 48 hour clamp tended to be greater (p = 0.059) in lean (58%) compared to obese subjects (44%).

### Munc18c and Syntaxin 4 Protein Content

**Figure 1:**
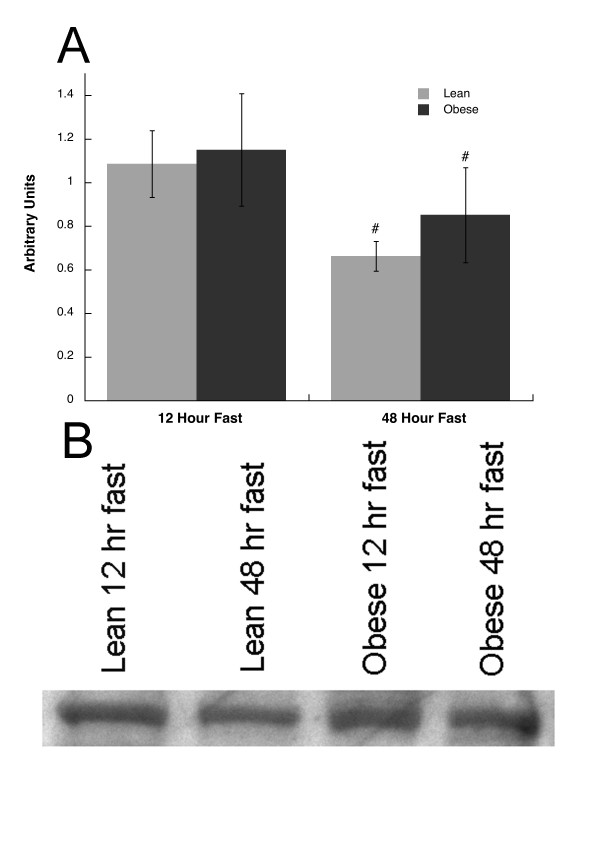
**A) Munc18c content after 12 and 48 hours of fasting in lean and obese subjects, B) Representative blot for Munc18c in lean and obese subjects.** Values are means ± SEM. # = significantly different than 12 hr (p < 0.05).

**Figure 2:**
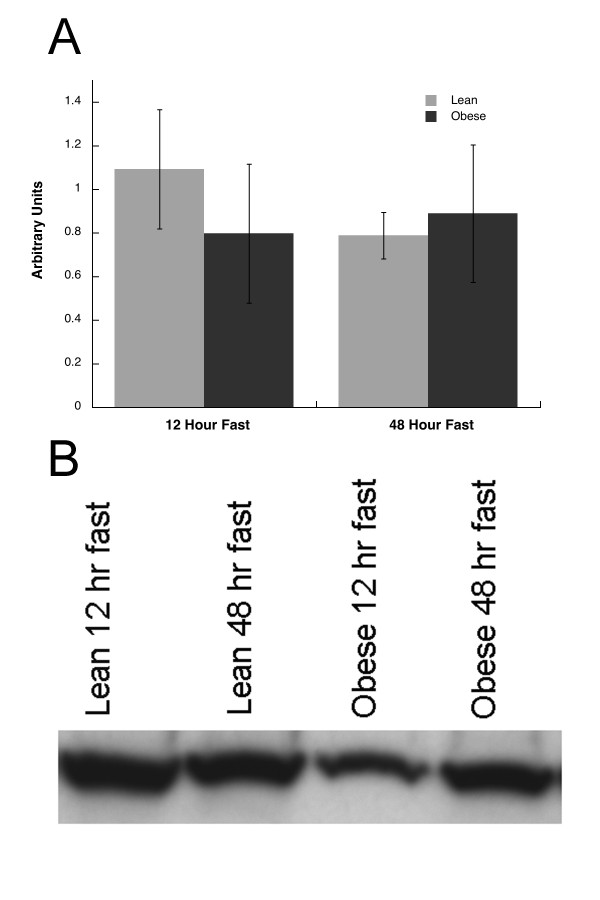
**A) Syntaxin 4 content after 12 and 48 hours of fasting in the basal state in lean and obese subjects, B) Representative blot for Syntaxin 4 in lean and obese subjects.** Values are means ± SEM.

No significant differences were observed for Munc18c content in skeletal muscle between lean and obese subjects after 12 or 48 hour fasts (Figure 1A). Munc18c content decreased in the lean and obese subjects after the 48 as compared to the 12 hour fast (p = 0.013, Figure 1A). Syntaxin 4 content was not different between lean and obese subjects, and did not significantly change after 48 compared to 12 hours of fasting (Figure 2A).

**Figure 3:**
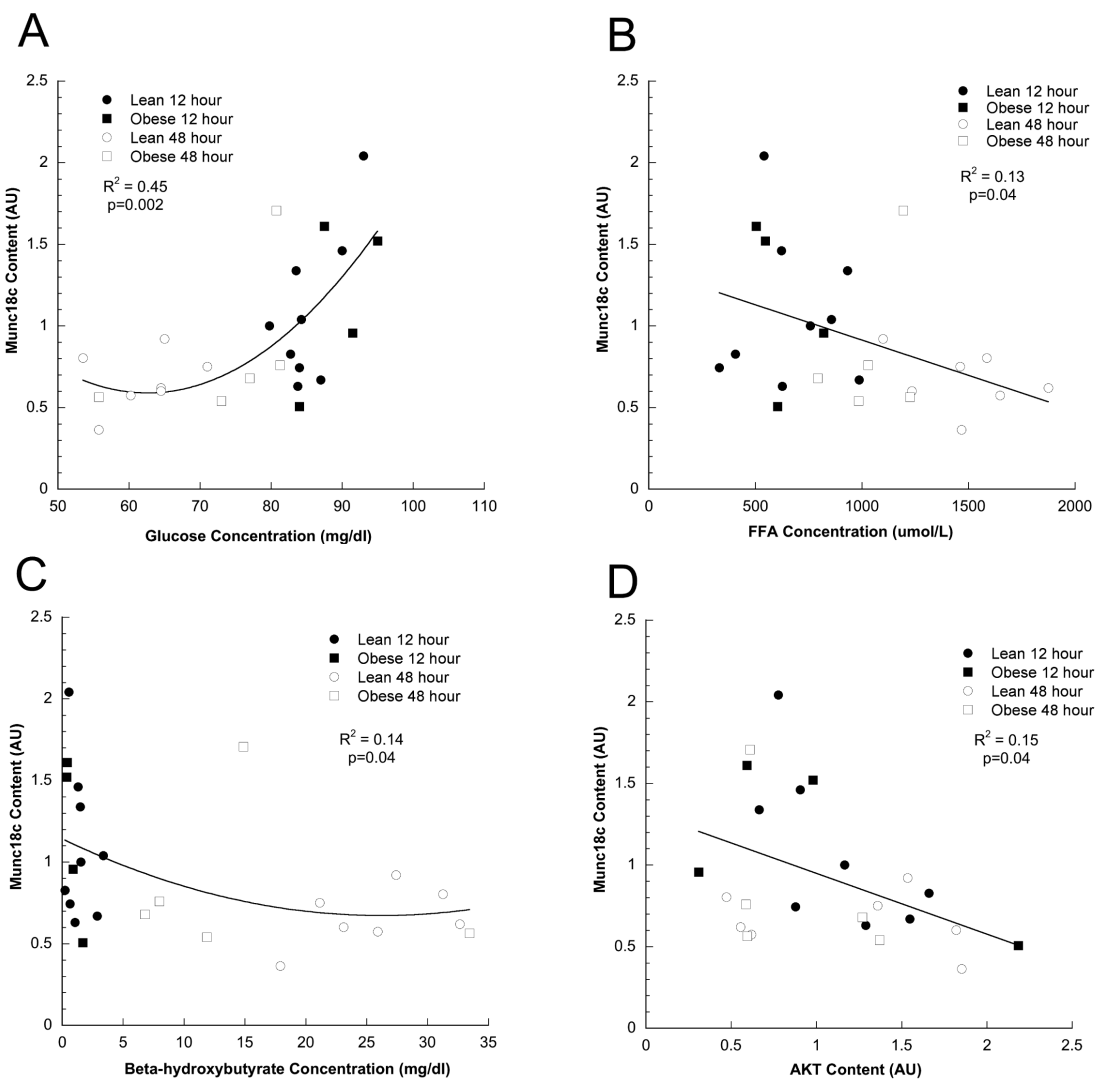
Relationships between skeletal muscle Munc18c content and A) plasma glucose (R^2 ^= 0.45, p = 0.002) B) plasma FFA (R^2 ^= 0.13, p = 0.04) C) plasma beta-hydroxybutyrate (R^2 ^= 0.14, p = 0.04) and D) skeletal muscle AKT content (R^2 ^= 0.15, p = 0.04) and in lean and obese men and women after 12 and 48 hours of fasting.

There was a strong significant positive relationship between plasma glucose concentration and muscle Munc18c content in lean and obese subjects following both 12 and 48 hr fasts (R^2 ^= 0.45, p = 0.002, Figure 3A). Several other relationships were discovered including significant negative correlations between muscle Munc18c and plasma FFA (Figure 3B, R^2 ^= 0.13, p = 0.04), serum beta hydroxybutyrate (Figure 3C, R^2 ^= 0.14, p = 0.04), and total muscle AKT content (Figure 3D, R^2 ^= 0.15, p = 0.04). Relationships were not significant between muscle Munc18c and serum insulin concentration (p = 0.22), adiponectin (p = 0.63), leptin (p = 0.43), glucose disposal during the euglycemic insulin clamp (p = 0.15), muscle insulin receptor phosphotyrosine content (p = 0.09), serine307 phosphorylation of IRS-1/total IRS-1 (p = 0.11), and pan85 content (p = 0.11)

## Discussion

This is the first study to report tissue Munc18c, and Syntaxin 4 protein content in humans. We found no significant differences in the content of either protein in obese compared to lean subjects after an overnight fast (Figures 1 and 2). Therefore, these data suggest insulin resistance associated with obesity in humans is not explained by constitutive differences in skeletal muscle Munc18c and Syntaxin 4 content.

Prolonged fasting induced a significant decrease in glucose uptake during the clamp in lean and obese subjects after the 48 compared to the 12 hour fast (Table 1). Fasting for 48 hours did not change Syntaxin 4 (Figure 2), but significantly decreased Munc18c content in lean and obese subjects (Figure 1). These data suggest prolonged fasting decreased basal skeletal muscle Munc18c content regardless of body type, which may be one mechanism by which fasting decreased insulin stimulated glucose uptake.

The optimum amount of skeletal muscle Munc18c to promote GLUT4 vesicle fusion is not known. However, it has been demonstrated that deletion of the Munc18c gene is lethal [19], and that too little or too much Munc18c can alter insulin sensitivity in animals [7-9,20], and cell culture [6,10-12]. Further, the content of Munc18c relative to Syntaxin 4 appears more important than the absolute content of each on whole body glucose disposal in mice [7]. This study elaborates on previous data and suggests that Munc18c, and not Syntaxin 4, appears to be the regulated of these two proteins in human skeletal muscle.

We have previously reported increased Munc18c with no change in Syntaxin 4 content in mice over expressing skeletal muscle lipoprotein lipase [20] which correlated with decreased insulin action following high fat feeding. The current data are different from that found in rodents, as both Munc18c and Syntaxin 4 content were unchanged in obese compared to lean humans after an overnight fast despite significant differences in glucose Rd during the clamp.

Moreover, our data demonstrating decreased skeletal muscle Munc18c content after a 48 hour fast are not what was expected from the animal literature where tissue Munc18c content is negatively related to insulin action [6,9-11]. Therefore, it is possible that decreased Munc18c is a compensatory response to maintain insulin action during a prolonged fast. These discrepancies in data in humans vs. mice suggest a species difference in the regulation of Munc18c.

Basal Syntaxin 4 content was not significantly different between lean and obese subjects after 12 and 48 hours of fasting. In mice, decreased Syntaxin 4 content has been associated with insulin resistance during a clamp [9] and following streptozotocin-induced diabetes [21]. Our data differ from animal studies and suggest Syntaxin 4 content may not be involved in short-term regulation of insulin action in humans, and may not be important to obesity-induced insulin resistance.

We found a significant relationship between plasma glucose concentration and Munc18c content (Figure 3A, p = 0.0015) in lean and obese subjects after 12 and 48 hr fasts. Whether glucose concentration influences Munc18c content or *vice versa *remains to be determined. However, our data indicate close regulation between these 2 parameters. In the fasting state FFA and beta-hydroxybutyrate concentrations change dramatically, and could reduce by an unknown mechanism the Munc18c content of skeletal muscle. Another possible explanation is that alterations in the expression of muscle Munc18c may promote changes in glucose concentration and skeletal muscle AKT content. Animal data suggest that Munc18c may promote insulin resistance [6,9-11]. The relationship between plasma glucose concentration and skeletal muscle Munc18c in this study is consistent with this idea. If Munc18c influences glucose concentration, what else besides FFA and beta-hydroxybutyrate drives changes in Munc18c content? Previous data from our laboratory indicated skeletal muscle LPL overexpression in mice increased Munc18c and intramuscular triglyceride content, along with a decrease in insulin action [20]. These animal data suggest increased storage of intramuscular triglyceride in muscle may be related to increased Munc18c content. In hindsight, measurement of intramuscular triglyceride concentration would have been informative in this study to determine if there is a relationship with muscle Munc18c content.

The significant relationships between glucose (Figure 3A), FFA (Figure 3B) and beta-hydroxybutyrate concentration (Figure 3C) suggest there are potential associations between plasma substrates and muscle Munc18c. The significant negative relationship between Munc18c and AKT content in skeletal muscle biopsies (Figure 3D) could indicate that increased Munc18c expression in skeletal muscle may be related to decreased AKT content, and promote insulin resistance. The relationship between Munc18c and AKT is somewhat puzzling because a decrease in Munc18c with fasting (Figure 1) corresponded to decreased insulin action (Table 1) as well as an increase in AKT content. However, we did not find a significant change in the phosphorylation of AKT relative to the total content, suggesting that the change in total content was not related to activity. Further, it is possible that both Munc18c and AKT content were altered in response to other signals, and that the change in one does not promote a change in the other. This study was not designed to test relationships between Munc18c and metabolic or insulin signaling variables. However, these relationships are intriguing and suggest that the content of Munc18c changes in parallel with blood metabolites and the insulin signaling cascade.

We are hesitant to discuss non-significant relationships, but given that this is the first report of Munc18c and Syntaxin 4 in humans, we feel obligated to show all data that may move the field forward if further studied. We did not find significant relationships between Munc18c and insulin concentration (p = 0.22), as well as the adipokines adiponectin (p = 0.63), and leptin (p = 0.43) which are thought to modulate insulin action [22]. We found several interesting, but non-significant negative relationships between Munc18c and glucose disposal during the insulin clamp (p = 0.15), and insulin signaling including insulin receptor phosphotyrosine content (p = 0.085), serine307 phosphorylation of IRS-1/total IRS-1 (p = 0.11), and pan85 content (p = 0.11). Given that there was a significant relationship between Munc18c and AKT content, these data suggest that there may be concurrent regulation of Munc18c and the insulin signaling cascade.

## Conclusion

These are the first data reporting Munc18c and Syntaxin 4 protein content in humans. No significant differences were observed for Munc18c and Syntaxin 4 content in lean and obese human skeletal muscle after 12 or 48 hours of fasting. However, along with a significant decrease in insulin sensitivity, Munc18c content decreased in lean and obese subjects after the 48 hour fast suggesting these two parameters may be related. We also found significant relationships between skeletal muscle AKT content, plasma glucose, FFA, and beta-hydroxybutyrate concentration and skeletal muscle Munc18c. These data suggest Munc18c is under short-term regulation and may influence insulin action in humans.

## Declaration of competing interests

The authors declare that they have no competing interests.

## Authors' contributions

BCB helped design the study, carried out all subject testing, ran all westerns, analyzed data, and wrote the manuscript. M–AC performed medical oversight, helped with subject testing and study design. TJH helped with subject testing, and helped design the study. DHB performed medical oversight, helped with subject testing and study design and provided scientific direction. RHE provided antibodies for the research, scientific direction, and helped write the manuscript. All authors read and approved the final manuscript.
